# Motivation for improving academic achievement in cosmetological education

**DOI:** 10.1002/hsr2.919

**Published:** 2022-10-29

**Authors:** Jooyoung Lee, Ki Han Kwon

**Affiliations:** ^1^ Department of Beauty Arts Care, Division of Beauty Arts Care, Graduate School Dongguk University Seoul Republic of Korea; ^2^ IJOO Co. Seoul Republic of Korea; ^3^ Department of Lifestyle Design, Division of Beauty Design, Graduate School of Professional Studies Sookmyung Women's University Seoul Republic of Korea; ^4^ College of General Education Kookmin University Seoul Republic of Korea

**Keywords:** academic achievement, beauty education, motivation learning, online learning, post‐COVID‐19

## Abstract

**Background and Objective:**

Before and after the fandom, the pattern of education has difficulty in accepting the requirements of the MZ generation with very intense changes. Individual differences between students and different educational environments have more difficulties with the addition of practical subjects than general students. In this study of the purpose, after coronavirus disease‐19 (COVID‐19), the change in demand was described for the factors that influence the motivation necessary in current beauty education for diversity in education methods of MZ generation.

**Methods:**

These thesis studies are literature reviews, and for this purpose, a narrative review approach was used. A total of 200–300 references were selected and reviewed, and a total of papers were finally selected as of 2010–2022 using the PRISMA flow diagram.

**Results:**

With the emergence of diversity in education, online education is showing its power. In beauty education, the need for education of practical subjects has emerged and is the basis for providing more skilled skills in the field. This review paper conducted a comprehensive study on the motivational factors that will be the basis for the improved academic achievement of the MZ generation on the changes in education after COVID‐19.

**Conclusion:**

This literature review revealed the needs of the subjects of K‐education to be seen after the post‐COVID era and the factors of learning motivation required in the beauty distance education of the MZ generation. In addition, the global status of K‐beauty workers has confirmed that new changes with beauty and intelligence are being born as well as simple technical, and it is expected to be used as an important material for beauty distance education.

## 1 INTRODUCTION

The Fourth Industrial Revolution has led to many developments affecting mankind. In the recent past, unimaginable things have happened, and various types of education and learner‐centered methods are being sought in response to such events. There are many factors that influence learning. In recent years, many social phenomena caused by the coronavirus disease‐19 (COVID‐19) pandemic have emerged that have led to changes throughout society.^1^ The MZ generation refers to the Millennium and Generation Z,^2^ which are generations before and after the Millennium, and are familiar with the online and digital environment, pursuing personal specifications that are distinct from others and optimized for the digital environments. This trend of social directions is inevitably confusing for students, and this is also the case in beauty education. In the social phenomenon of fandom post‐COVID‐19, the MZ generation has come to lead fashion in a number of major directions. It has become an old saying that good students have fallen behind in fashion. A student who is good at studying is good at everything and knows how to make themselves unique. In learning, psychological devices are invoked to present various directions along with the learner's will and to achieve the improved activity. Accordingly, the motivational element, which is the driving force for achieving that is necessary in learning, resonates very much, and research is actively being conducted, with prior researchers heavily emphasizing this point. Current research is examining academic achievement among students with achievement goal orientation, academic self‐efficacy, and self‐directed learning, which are the driving forces.^3^

In fact, the rate of university attendance among Korean high school students is the highest among Organization for Economic Cooperation and Development countries. Academic performance is very important for high school students to enter higher education. This is because Korean society determines the status of college and the level of university based on high school grades and the results of college entrance examinations, and it also has a number of effects on an individual's entry into society. In the midst of this, the problem of improving academic achievement came to the fore in beauty study, and factors related to academic motives began to be mentioned. Therefore, high school students strive to increase their academic achievement to enter the university of their choice.^4^ Therefore, the Ministry of Education is also carrying out various efforts to improve the academic achievement of students. The Ministry of Education annually assesses students' national‐level academic achievement to identify trends in changes in students' academic achievement and provide evidence for improving the curriculum as well as the teaching and learning methods. However, these efforts place too much emphasis on the effectiveness of objectivity assessment, which is a function of academic achievement, and are rather biased toward intellectual and educational outcomes.^5^, ^6^

Moreover, most students have been found to learn compulsorily and involuntarily rather than on their own. It is very important for students to clearly establish their own achievement goals and learn to achieve them. In other words, it is very important for learners to be goal‐oriented to realize their dreams, even in the face of substantial changes occurring in different environments.^7^ In addition, academic self‐efficacy is also important for students because it enhances self‐efficacy in their studies. In particular, in online education, self‐directed learning ability is the ability of learners to plan, practice, and evaluate their own learning as an important inventory. Therefore, it is predicted that the study of academic achievement through academic motivation in beauty major education will act as an important factor in preparing for future learning.

## 2 MATERIALS AND METHODS

**Figure 1:**
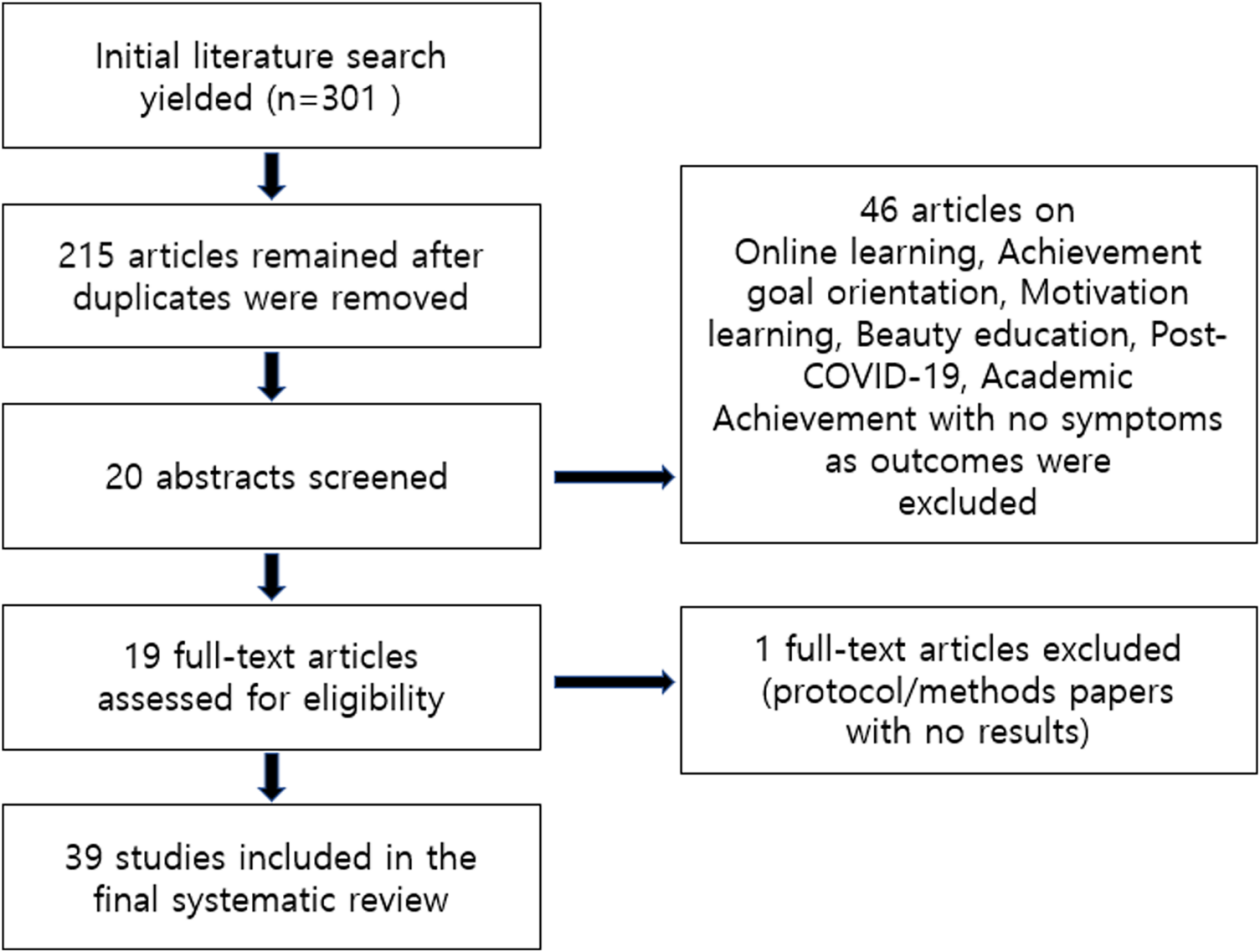
Flow of literature search using PRISMA

These studies are literature reviews, and for this purpose, a narrative review approach was used. A total of 200–300 references were selected and reviewed, and the reason for using previous studies during this period from 2010 to 2022 reflects relatively recent research trends. Achievement goal orientation, motivation learning, beauty education, post‐COVID‐19, results in 40 asymptomatic academic achievements, qualification evaluation, 39 papers, excluding 1 expert articles (protocol/method papers without results, 1 expert articles) have been finalized. This was shown using PRISMA diagram (Figure 1).

## 3 RESULTS

### 3.1 The relationship between achievement goal orientation that motivates learning, academic self‐efficacy, self‐directed learning ability, and academic achievement and beauty education

This is related to achievement goal orientation, which is the result of the learner's own belief in learning and willingness to pursue academic achievement along with their learning goal.^5^, ^8^ Moreover, goal orientation increases academic achievement, and direct intervention in learners' achievement behavior influences the achievement of learning goals and the path to academic achievement.^9^ Further, it synchronizes the use of learning strategies with the learning process related to academic achievement to ensure that the tasks are solved smoothly. In other words, goal‐oriented learners value academic outcomes and demonstrate a high sense of achievement through the use of active learning strategies and academic effort.^10^ Direct intervention in learners' achievement behavior also influences the achievement of learning goals and the path to academic achievement.^9^ Further, it synchronizes the use of learning strategies with the learning process related to academic achievement to ensure that the tasks are solved smoothly. Therefore, to improve academic achievement and accomplish achievement goals, learners must be able to develop and implement career plans on their own.^6^ This sense of academic self‐efficacy is more densely defined by the syllabus than the self‐efficacy of the overall study, which affects academic achievement.^11^ In other words, academic self‐efficacy is more closely related to the results of academic achievement according to the syllabus than it is to the general self‐efficacy of the discipline as a whole.^12^, ^13^ Students with a high degree of academic self‐efficacy tend to identify a lack of coursework on their own and then engage in prior learning or review.^14^ The learner's ability to engage in self‐directed learning in a variety of academic settings is very important.^15^ A learner with high self‐directed learning ability actively participates in learning activities and acts in a goal‐oriented manner, and it is important to emphasize the independence of the learner.^15^ It is important for learners to have self‐directed learning skills to learn effectively and obtain academic achievements.^16^ While self‐directed learning skills are found to be increasingly important, the self‐directed learning attitude among Korean students is still overall lower than those of other countries. As a result, it is important to conduct research to improve self‐directed learning ability, and it can be said that there is a need for studies examining the relationship between self‐directed learning ability and academic achievement in high school students.^17^, ^18^, ^19^ Beauty education is also subject to various motivational factors and studying to make people beautiful is a new creative job involving the human body, so there is a possibility of infinite development along with online education in the future. Table 1 is presented to help understanding of these contents.

**Table 1:** Relationship between achievement goal orientation, academic self‐efficacy, self‐directed learning ability, and academic achievement

No	Journal name	Authors	Title	Discussion	Reference		
1	*Spanish Journal of Psychology*	Abd‐El‐ Fattah (2010)^16^	Garrison's model of self‐directed learning: preliminary validation and relationship to academic achievement	Influence of self‐directed learning and academic achievement in classroom environment by using psychological factors and motivation.	[16]		
2	*International Education Studies*	Saeid and Eslaminejad (2017)^15^	Relationship between student's self‐directed‐learning readiness and academic self‐efficacy and achievement motivation in students	Presenting a strategy of self‐directed learning to improve academic achievement through learning motivation.	[15]		
3	*International Journal of Research in Education and Science*	Kotluk and Kocakaya (2017)^18^	The effect of creating digital storytelling on secondary school students' academic achievement, self efficacy perceptions and attitudes toward physics.	Self‐efficacy presents the importance of motivation through differences in physics academic achievement.	[18]		
4	*European Journal of Educational Research*	Rabia et al. (2018)^9^	Prospective preschool teachers' academic achievements depending on their goal orientations, critical thinking dispositions and self‐regulation skills	Contribution to the development of orientation through the influence of preservice teachers' goal orientation on academic achievement.	[9]		
5	*Educational Psychology Review*	Morris et al. (2017)^20^	Reconceptualizing the sources of teaching self‐efficacy: a critical review of emerging literature	Through 82 experience studies of teachers, teachers' efficient beliefs and motivations affect students' academic achievement.	[20]		

### 3.2 Relationship between learning motivation factors in theory and practice in beauty education and online education

Until now, research on academic achievement has shown that cognitive factors such as intellectual potential, academic ability, and intelligence are deeply related to students' academic achievement. In addition to cognitive factors, it has been suggested that noncognitive factors such as learners' motivation, emotions, personality, home environment, school environment, and efficacy are also relevant parts of students' academic achievement.^21^ In this regard, it would be meaningful to look at the noncognitive factors that influence academic achievement: specifically, academic self‐efficacy and self‐directed learning ability. However, much of the existing research on achievement goal orientation has mainly considered college students, and there has been relatively little research on adolescents.^22^ In addition, achievement goal orientation is largely based on cognitive factors for achieving goals for career or academic purposes. In addition, in the current post‐COVID‐19 era, it can lead to the activation and connection of online and practical offline classes of beauty education theory subjects for students in the beauty department.^23^, ^24^, ^25^ Although some studies related to achievement goal orientation have dealt with noncognitive factors, such as achievement sentiment, and self‐leadership self‐determination, this area is still lacking. On the other hand, there have been many studies analyzing the impact of academic self‐efficacy on academic achievement and career decisions.^26^ Among these many prior studies, there has been research examining the relationship between academic self‐efficacy and academic achievement, but few studies have been simultaneously considered academic self‐efficacy, achievement goal orientation, and self‐directed learning ability.^21^ Beauty and cosmetological education involves completing academic courses in the process of linking practical education with theoretical studies, is recognized in the actual beauty industry, shortens the internship process by reducing the long apprenticeship period, and leads to the achievement of real NCS education in the beauty industry, the showing at Table 2.

**Table 2:** Relationship between learning motivation factors in online beauty education

No	Journal name	Authors	Title	Discussion	Reference		
1	*Education and Information Technologies*	Parmaksız (2022)^19^	The mediating role of personality traits on the relationship between academic self‐efficacy and digital addiction.	Effects of 876 women and 472 men in Turkish university students on learning motivation factors of university students	[19]		
2	*Vocational Education International Conference*	Widowati et al. (2019)^27^	Analysis of practical assessment sheet needs in beauty education programs in state university	Overall understanding of the program on beauty education for college students	[27]		
3	*Springer*	Yokoyama and Miwa (2020)^28^	Relationship between goal orientation, conception of learning, and learning behavior, in online teaching and learning in higher education.	Understanding goal orientation in online education and learning and emphasizing the importance of the role of achievement motivation in online education	[28]		
4	*Journal of the Korean Society of Cosmetology*	Cho and Hyeok (2016)^24^	A study on the awareness of Korean cosmetics and interest of Korean beauty education from mongolia, vietnam and china.	Online and practical offline classes of beauty education theory subjects of students in the beauty department in response to the post‐COVID‐19 era are activated	[24]		
5	*Journal Pendidikan Teknologi Kejuruan*	Flowriza and Rahmiati (2021)^29^	Evaluation of implementation of learning practices during pandemic Covid‐19 coating and beauty education study program.	Study 104 students with makeup during the Covid‐19 pandemic discuss the interpretation of the product in the education program	[29]		

### 3.3 Comprehensive art of self‐directed learning, continuous esthetic evaluation and emotional response, the future of beauty education

Cosmetological education is a comprehensive art that involves decorating the human body, and unlike other education, it is an esthetic concept that includes the expression of inner beauty and extroverted beauty with the aim of decorating the human body comprehensively, not just one part. Famous artists also explain beauty as a job that allows for expressions of applied art.^30^ For example, there are studies based on beauty works that derive ideas from Antonio Gaudi's architectural works to create works.^31^ Beauty art is an applied art, and it has the importance of understanding and touching human psychological functions; it is an art that grows in humans. Learner's self‐directedness is important, as it serves as the basis of original artwork and leads to a higher level of beauty education. This review of research related to self‐directed learning skills has been researched in various studies, mainly among elementary, middle, high, and college students.^32^, ^33^ However, self‐directed learning skills are mostly based on learning ability, educational beliefs, leadership, autonomy, problem‐solving ability, and learning satisfaction.^34^ In addition, although there have been studies related to self‐directed learning skills and academic achievement, there has been insufficient research on primary school and college students. Over the years, a variety of studies have been conducted by many researchers on factors such as achievement goal orientation, academic achievement, academic self‐efficacy, and self‐directed learning ability.^3^ However, in previous studies, academic self‐efficacy has been treated as a mediator factor in the relationship between parents' parenting attitudes and academic achievement, and self‐directed learning ability has been found to have a mediating effect in the relationship between emotions, learning satisfaction, learning methods, and academic achievement.^35^ In this respect, research that identifies the mediating effects of academic self‐efficacy and self‐directed learning ability in the structural relationship in which achievement goal orientation affects academic achievement is very significant. In other words, it is very important to conduct research examining the effects of self‐directed learning ability, which plays an important role in learning planning, learning implementation, and learning evaluation in learners' academic commitments, along with the effect of academic self‐efficacy on academic achievement that drives learners' learning to higher levels and balanced performance.^36^ This is because it creates an autonomous learning environment for learners and provides them with effective individual learning methods. Therefore, compared to these various prior studies, this study can be differentiated as follows (Table 3).

**Table 3:** Comprehensive art of self‐directed learning

No	Journal name	Authors	Title	Discussion	Reference		
1	*Brain Science Research*	Song and You (2011)^3^	The effect of psychological factors and family environment variables on academic achievement: focusing on high school students in humanities	Psychological factors of high school students and environmental influences on academic achievement.	[3]		
2	*The Caribbean Teaching Scholar*	Bodkyn and Stevens (2015)^36^	Self‐directed learning, intrinsic motivation and student performance.	Self‐directed learning and intrinsic motivation of 485 medical students bring emotional stability, so positive outcomes for students' academic achievement.	[36]		
3	*International Journal of School & Educational Psychology*	Schweder (2019)^35^	The role of control strategies, self‐efficacy, and learning behavior in self‐directed learning	The mediating role of the control strategy has the psychological stability of adolescents, resulting in an increase in self‐efficacy.	[35]		
4	*International Journal of Instruction*	Sukardjo et al. (2020)^33^	Effect of concept attainment models and self‐directed learning (SDL) on mathematics learning outcomes	In the math learning results, it is easy for students with low SDL with a high psychological anxiety index to achieve the concept in comparison with the self‐directed learning model	[33]		
5	*Poetics*	Wagner et al. (2021)^30^	Effects of continuous self‐reporting on aesthetic evaluation and emotional responses	In the group comparison for empirical esthetics, the psychological and physical response of subjective experience is the subjective experience system no change through the heart rate check.	[30]		

## 4 DISCUSSIONS

As seen above, in future beauty education, not just cramming education, but various visual elements should be added. As a result, academic achievement, which is the result of academic work, will also lead to achievement education in the future and will bring about environmental changes. Education in the classroom is likely to take many different forms with COVID‐19 as a turning point. In the form of education before and post‐COVID‐19, there is an emerging need for metaverse‐style virtual space and education without space restrictions in the classroom. The development of science is breaking its limits. The future we have dreamed of in which we can do everything is becoming a reality, and education is moving in the same direction. We are in an era where everything from school to meeting teachers, meeting friends, and studying is now possible online. To move forward in social pathology, we need to study the steps that must be taken to avoid any negative effects of this social phenomenon. Studying is both a duty and a responsibility, and it is also a great opportunity to prepare for the future.^37^, ^38^

As beauty and cosmetological education is also practical subject, repeated practice is needed to acquire skills. It is very important for such technical education to be taught closely by the teacher. However, there is an environmental challenge, and students need it very much. If that is the case, iterative learning and real‐time learning must be compatible through online education. Accordingly, online education leads to much improved academic achievement through the synergy of education with students' learning motivation.^30^ In other words, beauty education, unlike other education, is education that combines theory and practice, and the improvement of academic motivation and academic achievement accordingly is very important. Therefore, students have no choice but to suffer from double whammy, and to solve this problem, they should have a clear achievement orientation based on emotional stability and come up with ways to improve academic achievement that clearly affects future jobs. In beauty education, the need for real‐time education and repeated learning of online education based on these futuristic online technologies, that is, metaverse technology and future technologies such as virtual reality should be embraced. The world is now in an era where everyone can gather together immediately.^38^, ^39^ The Korean beauty industry will now continue improving through online beauty education. Just as the world was enthusiastic about the K‐pop superstar's‐Bang Tan Soyeundan, Korea is posed to make a leap forward as a mecca for the beauty industry, as it was before COVID‐19.

## 5 CONCLUSIONS

Through this literature review as an important achievement goal orientation, academic self‐efficacy, and self‐directed learning ability, which are learning motivation factors, which are important variables for improving academic achievement in remote beauty education according to the post‐COVID‐19 era, were examined in beauty education, focusing on the MZ generation. Learning motivation plays an important role in improving academic achievement. Specially, learning motivation in beauty education is online learning through future augmented reality, and it was confirmed that achievement goal orientation, self‐directed learning ability, and academic self‐efficacy play an important role in learning. Among them, it was thought that self‐directed learning ability to plan and practice self‐learning in online education would be a very important factor. In addition, the global status of K‐beauty workers confirmed that new changes are taking place through beauty and intelligence along with simple technological changes and is expected to be used as important data for beauty distance education. In the future, through quantitative research on factors that have an important influence on academic achievement improvement in beauty education, research on personal and environmental factors necessary to improve academic achievement in beauty education is suggested.

## AUTHOR CONTRIBUTIONS

**Jooyoung Lee**: Conceptualization. **Ki Han Kwon**: Supervision.

## CONFLICT OF INTEREST

The authors declare no conflict of interest.

## ETHICS STATEMENT

The conducted literature review did not require the agreement of the bioethics committee.

## TRANSPARENCY STATEMENT

The lead author Ki Han Kwon affirms that this manuscript is an honest, accurate, and transparent account of the study being reported; that no important aspects of the study have been omitted; and that any discrepancies from the study as planned (and, if relevant, registered) have been explained.

## Data Availability

The findings of this study are available from the corresponding author upon reasonable request.
